# Comparative analysis of the expression level of recombinant ginsenoside-transforming *β*-glucosidase in GRAS hosts and mass production of the ginsenoside Rh_2_-Mix

**DOI:** 10.1371/journal.pone.0176098

**Published:** 2017-04-19

**Authors:** Muhammad Zubair Siddiqi, Chang-Hao Cui, Seul-Ki Park, Nam Soo Han, Sun-Chang Kim, Wan-Taek Im

**Affiliations:** 1 Department of Biotechnology, Hankyoung National University, Kyonggi-do, Republic of Korea; 2 Center for Genetic Information, Graduate School of Bio and Information Technology, Hankyoung National University, Kyonggi-do, Republic of Korea; 3 Intelligent Synthetic Biology Center, Yuseong-gu, Daejeon, Republic of Korea; 4 Brain Korea 21 Center for Bio-Resource Development, Division of Animal, Horticultural and Food Sciences, Chungbuk National University, Cheongju, Korea; Macau University of Science and Technology, MACAO

## Abstract

The ginsenoside Rh_2_, a pharmaceutically active component of ginseng, is known to have anticancer and antitumor effects. However, white ginseng and red ginseng have extremely low concentrations of Rh_2_ or Rh_2_-Mix [20(*S*)-Rh_2_, 20(*R*)-Rh_2_, Rk_2_, and Rh_3_]. To enhance the production of food-grade ginsenoside Rh_2_, an edible enzymatic bioconversion technique was developed adopting GRAS host strains. A β-glucosidase (BglPm), which has ginsenoside conversion ability, was expressed in three GRAS host strains (*Corynebacterium glutamicum*, *Saccharomyces cerevisiae* and *Lactococus lactis*) by using a different vector system. Enzyme activity in these three GRAS hosts were 75.4%, 11.5%, and 9.3%, respectively, compared to that in the *E*. *coli* pGEX 4T-1 expression system. The highly expressed BglPm_C in *C*. *glutamicum* can effectively transform the ginsenoside Rg_3_-Mix [20(*S*)-Rg_3_, 20(*R*)-Rg_3_, Rk_1_, Rg_5_] to Rh_2_-Mix [20(*S*)-Rh_2_, 20(*R*)-Rh_2_, Rk_2_, Rh_3_] using a scaled-up biotransformation reaction, which was performed in a 10-L jar fermenter at pH 6.5/7.0 and 37°C for 24 h. To our knowledge, this is the first report in which 50 g of PPD-Mix (Rb_1_, Rb_2_, Rb_3_, R_c_, and Rd) as a starting substrate was converted to ginsenoside Rg_3_-Mix by acid heat treatment and then 24.5-g Rh_2_-Mix was obtained by enzymatic transformation of Rg_3_-Mix through by BglPm_C. Utilization of this enzymatic method adopting a GRAS host could be usefully exploited in the preparation of ginsenoside Rh_2_-Mix in cosmetics, functional food, and pharmaceutical industries, thereby replacing the *E*. *coli* expression system.

## Introduction

The ginseng (*Panax ginseng* C.A. Meyer) is a famous herbal medicinal plant, which is broadly circulated in Asian and Western countries and used for millions of years for the cure of human diseases [1]. For the last decades, especially, red ginseng is used as a common tonic for its high pharmacological activities. The biological and pharmacological active components of *P*. *ginseng* are commonly known as ginsenosides (major ginsenosides), a class of triterpene glycosides [2–4].

The major ginsenosides, PPD-type [protopanaxadiol type (Rb_1_, Rb_2_, Rb_3_, Rc and Rd)] and PPT-type [protopanaxatriol type (Re, and Rg_1_)] are present more than 90% of all ginsenosides, in the ginseng constitute [2, 5]. But, due to its high molecular weight the absorption of these major ginsenosides are very difficult through by the human digestive tract system [6, 7]. Therefore, these major ginsenosides are converted into minor ginsenosides by using of various methods including physical (heat treatment), chemical (acid or base treatment) and biological (microorganisms or enzymes) transformation. The minor ginsenosides (including, F_1_, F_2_, Rg_2_, Rg_3_, Rh_1_, Rh_2_ and C-k) which are the de-glycosylated byproducts from major ginsenosides are present in smaller amounts in the ginseng extract or powder. These minor ginsenosides show high pharmacological effects for anticancer, anti-allergy, anti-inflammatory, antitumor, antidiabetic, and anti-osteoporosis effects [8–10] than major ginsenosides.

In particular, the minor ginsenoside Rh_2_ can inhibit the growth of many kinds of cancer cells, including breast cancer, prostate cancer, hepatoma, gastric cancer, colon carcinoma, and pancreatic cancer; moreover, pre-clinical assessment of Rh_2_ in the PC-3 human xenograft model for prostate cancer in *vivo* has also been shown to be effective [11–17]. In addition, Rh_2_ also inhibits osteoclastogenesis [18], induces the differentiation and mineralization of osteoblastic MC3T3-E1 cells through activation of PKD and p38 MAPK pathways [19], improves learning and memory [20], reduces cell proliferation, and increases sub-G1 cells [21]. Furthermore, ginsenoside Rh_2_ improves the scopolamine-induced learning deficiency in mice [22],increases secretion of insulin and lowers plasma glucose in Wistar rats [23], has an antiobesity effect related to the activation of adenosine monophosphate (AMP)-activated protein kinase (AMPK) signaling pathway in 3T3-L1 adipocytes [24], and dose-dependently decreases the acanthosis and papillomatosis index, T lymphocyte percentage, and vessel density in PN skin grafts in mice [25].

The total amount of minor ginsenosides is much less in ginseng extract/powder; researchers are therefore interested in scaling up the production of minor ginsenosides for commercial use in both herbal medicine and food supplementary products. In the early stages of this research, Bae et al. studied the conversion of ginsenosides in the human gastrointestinal tract by gut microorganisms [26]. Thereafter, a highly active recombinant glycoside hydrolase belonging to family I and family III was introduced for the conversion of major ginsenosides into minor ginsenosides for their pharmacological and cosmetic applications [27–32].

Initially, this ginsenoside-transforming glycoside hydrolase was mostly expressed in *Escherichia coli* and no researchers had yet studied the expression of the *β*-glycoside hydrolyzing gene in a GRAS (generally recognized as safe) host strain for the purpose of converting major ginsenosides into minor ginsenosides for mass production. Recently, Li *et al*. performed the conversion of major ginsenosides into minor ginsenosides using an expression system of *Lactococcus lactis*. The GRAS host organisms are non-pathogenic and recommended as food safe [33]. Compared to pathogenic strains, expression of the ginsenoside-transforming *β*-glucosidase in these GRAS strains is advantageous and of considerable interest for the commercial production of pharmacologically active minor ginsenosides and food supplements (i.e., industrial demand).

In the present study, the expression levels of recombinant ginsenoside-transforming *β*-glucosidase, from *Paenibacillus mucilaginosus* [27], were compared between GRAS hosts strains and *E*. *coli*. After remarkable successes with GRAS host expression (*Corynebacterium glutamicum*, *Saccharomyces cerevisiae*, and *Lactoccocus lactis*), the 24.5 g scale-up production of Rh_2_-Mix [20(*S*)-Rh_2_, 20(*R*)-Rh_2_, Rk_2_, Rh_3_] was performed from Rg_3_-Mix [20(*S*)-Rg_3_, 20(*R*)-Rg_3_, Rk_1_, Rg_5_] using the highly expressed enzymes in *C*. *glutamicum*. To our knowledge, this is the first report of scale-up production of high-value Rh_2_-Mix, using combined methods of acid treatment and food-grade recombinant enzymes expressed in GRAS host strain *C*. *glutamicum*. The results of this study would likely broaden the application of ginsenoside Rh_2_ and Rh_2_-Mix in the cosmetic, functional food, and pharmaceutical industries, thereby replacing the *E*. *coli* expression system.

## 2. Materials and methods

### 2.1. Materials

Ginsenosides standards, Rb_1_, Rc, Rb_2_, Rd, 20(*S*)-Rg_3_, 20(*R*)-Rg_3_, 20(*S*)-Rh_2_, F_2_ and C-K, were bought from Nanjing Zelang Medical Technology Co., Ltd. (China), while ginsenosides 20(*R*)-Rh_2_, Rk_1_, Rg_5_, Rk_2_ and Rh_3_ were purchased from Chengdu Biopurify Phytochemicals Co., Ltd. (China). The PPD-Mix type ginsenosides mixture from the root of *Panax quinquefolius* [American root saponins, mainly contained Rb_1_ (328 mg/g), Rc (173 mg/g), Rd (107 mg/g) and small amounts of Rb_2_ (25 mg/g) and Rb_3_ (25 mg/g)] acquired from Hongjiou Biotech Co. Ltd. (China) was used as the initial substrate in the current investigation. The genomic DNA from *Paenibacillus mucilaginosus* KCTC 3870^T^, *E*. *coli*, and pGEX 4T-1 plasmid (GE Healthcare, USA) were used for the *β*-glucosidase gene, host, and expression vector sources, respectively. *P*. *mucilaginosus* KCTC 3870^T^ was grown in aerobic conditions at 37°C on nutrient agar (NA, BD, USA). The recombinant *E*. *coli* for protein expression was cultivated in a Luria-Bertani (LB) medium supplemented with ampicillin (100 mg/l). *C*. *glutamicum* and the pCES208 plasmid, *S*. *cerevisiae* and pYES 2.1 plasmid, *L*. *lactis* strain NZ9000 and PNZ8148 plasmid (MoBiTec GmbH, Germany) were used as host, and expression vector sources, respectively (Table 1).

**Table 1:** Bacterial strains and plasmids used in this study.

Hosts	Relevant genotype or description	Sources or references
BL21 (DE3)	fhuA2 [lon] ompT gal (λ DE3) [dcm] ΔhsdS λ DE3 = λ sBamHIo ΔEcoRI-B int::(lacI::PlacUV5::T7 gene1) i21 Δnin	NEB strain catalog no. C2527
BL21(DE3) harboring pGEX-*BglPm*	Cloned with glucoside hydrolyzed (BglPm) gene for ginsenosides transformation	[27]
*C*. *glutamicum* ATCC 13032	Biotin-auxotrophic wild type	ATCC
*C*. *glutamicum* WJ001	ATCC 13032, cloning host for ginsenosides transformation	This study
*S*. *cerevisiae* CEN.PK113-5D	MATa *ura3-52 MAL2-8*^*c*^ *SUC2*	[37, 38]
*S*. *cerevisiae* CEN.PK113-5D	Cloning host for ginsenosides transformation	This study
*L*. *lactis* pNZ8148	Cloning host for ginsenosides transformation	MoBiTec
**Plasmids**		
pGEX-*BglPm*	Harboring β-glucosidase (BglPm) gene	[27]
pCES208	*E*. *coli*/*C*. *glutamicum* shuttle vector, 5.93 kb; Kan^R^	[39]
pCES208	Expression vector for His-tag fusion in *C*. *glutamicum* ATCC 13032 with *β*-glucosidase (BglPm) gene; Kan^R^	This study
pYES2	Ap^R^ *URA3 GALp*	Invitrogen Corporation
pYES2.1	pYES2.1 TOPO^®^ TA vector	[40]
pYES2.1	Expression vector for BglPm gene in *S*. *cerevisiae* 1389	This study
pNZ8148	Broad-host-range vector; *Cm*^*R*^, *PnisA*	MoBiTec
pNZ8148	Expression vector for BglPm gene in *L*. *lactis*	This study

CECT, Coleccioèn Espanìola de Cultivos Tipo; YGSC, Yeast Genetic Stock Center, Berkeley, CA, USA.

Kan^R^, kanamycin resistance

Cm^R^, chloroampinicol resistance

### 2.2. Rg_3_-Mix preparation as substrate

The ginsenosides Rg_3_-Mix [20(*S*)-Rg_3_ (118.6 mg/g), 20(*R*)-Rg_3_ (108.8 mg/g), Rk_1_ (144.9 mg/g), and Rg_5_ (170.5 mg/g)] was prepared from PPD-Mix using heat treatment with organic acid. The PPD-Mix was dissolved in distilled water at a concentration of 50 g/l and included citric acid (2%, w/v) and heat-treated (121°C for 15 min). After the reaction, resultant Rg_3_-Mix was used as the substrate for the subsequent enzyme reaction.

### 2.3. Molecular cloning, expression, and purification of recombinant BglPm in GRAS

The genomic DNA from *Paenibacillus mucilaginosus* KCTC 3870^T^ was extracted using a genomic DNA extraction kit (Solgent, Korea). The gene encoding *β*-glucosidase (*BglPm*) [27], which has ginsenoside-transforming activity, was amplified from the genomic DNA as a template via a polymerase chain reaction (PCR) using *Pfu* DNA polymerase (Solgent, Korea). The sequence of the oligonucleotide primers used for the gene cloning was based on the DNA sequence of BglPm (*β*-glucosidase; GenBank accession number: **AEI42200**). Four sets of primers (Table 2) were designed and synthesized by Macrogen Co. Ltd. (Korea) to amplify the gene of BglPm for *E*. *coli* and three kinds of GRAS strains. The amplified DNA fragment obtained from the PCR was purified and inserted into the pGEX 4T-1 GST fusion vector, pYES2.1 His-tag combined vector, pCES208 Histag combined vector, and pNZ8148 vector, respectively, using an EzCloning Kit (Enzynomics Co. Ltd., Korea). The resulting recombinant pGEX-BglPm, pYES2.1-BglPm, pCES208-BglPm, and pNZ8148-BglPm were transformed into *E*. *coli* BL21 (DE3), *C*. *glutamicum*, *S*. *cerevisiae*, and *L*. *lactis* strain, respectively. The bacterial strains and plasmids used in this study, their relevant characteristics, and their sources or references are given in Table 1.

**Table 2:** Primers used in this study (sequences 5′→3′).

Function and primer	Sequence
GST-pGEX 4T-1 fusion construction	
pGEX 4T-1-F pGEX 4T-1-R	G GTT CCG CGT GGA TCC GAA TAT ATT TTT CCA CAG CAA TTT GATG CGG CCG CTC GAG TTA CAG CAC TTT CGT GGA TGC GAT
His tag- pCES208 and pYES2.1 fusion construction	
pCES208-F pCES208-R pYES2.1-F pYES2.1-R	GACTAGAGTCGGATCC ATG GAATATATTTTTCCACAG CCGCGGTGGCGGCCGC TTA CAGCACTTTCGTGGATGC TATTAAGCTCGCCCTTATGGAATATATTTTTCCACAGCAATTT CTCGAAGCTCGCCCTTTTACAGCACTTTCGTGGATGC
*β*-glucosidase fusion construction	
pNZ8148-F pNZ8148-R	GCAGGCATGCGGTACCATG GAATATATTTTTCCACAG GCTTGAGCTCTCTAGATTA CAGCACTTTCGTGGATGC

### 2.4. Comparison of expressed enzyme activity in GRAS host

The *E*. *coli* strain BL21, and the three GRAS hosts strains were constructed with different vector systems —pGEX 4T-1, pCES208, pYES 2.1 and pNZ8148, respectively. To determine the levels of expression and the amount of soluble protein, the induction of expression of recombinant *E*. *coli* and three GRAS hosts studied was performed. The recombinant *E*. *coli* was cultivated in LBA (Luria-Bertani with ampicillin [100 mg/l final concentration]) and induced by 0.15 mM IPTG at 28°C. Similarly, *C*. *glutamicum*, *S*. *Cerevisiae*, and *L*. *lactis* were cultivated in LBK (Luria-Bertani with kanamycin [50 mg/l final concentration] induced by glucose [10 g/l final]), YPD (galactose inducible [18 g/l final concentration]), and GM–17 [glucose 10 g/l and induced by nisin, 10 μl/l final concentration)] at 30°C, respectively. After maximum growth of recombinant strains in the mentioned media, the cells were collected and sonicated for comparative analysis of their enzyme activity.

#### 2.4.1. Effect of sonication on enzyme activity of both recombinant *E*. *coli* and GRAS hosts

After the exponential growth of the strains in the particular media as stated above, cells were harvested by centrifugation and pellets were washed twice with a solution consisting of 100 mM sodium phosphate buffer and 1% Triton X-100 (pH 7.0); cells were then resuspended to a concentration of 1 g/10 mL in cold lysis buffer (100 mM sodium phosphate buffer [pH 7.0]). The crude cell extracts were obtained by sonication of the cell pellets using Branson digital sonifier 450 (400 W, 70% power, USA). Total sonication time was 20 and 30 min for *E*. *coli* and GRAS hosts strains respectively. After each 2 and 5 min interval, cell lysates were collected in a 1.5 ml tube in order to check enzyme activity and the effect of sonication with time intervals.

#### 2.4.2. Comparison of crude enzymes activities of GRAS host strains with recombinant *E*. *coli*

The activity of crude recombinant *β*-glucosidase was determined using 5 mM pNPGlc (p-nitrophenyl-*β*-D-glucopyranoside) as substrate. Crude enzyme (20 μL) was incubated in 100 μL of 50 mM sodium phosphate buffer (pH7.0) containing 5 mM PNPGlc at 37°C, then the reaction was stopped by 0.5 M (final concentration) Na_2_CO_3_ and the release of p-nitrophenol was measured immediately using a microplate reader at 405 nm (Bio-Rad Model 680; Bio-Rad, Hercules, CA). One unit of activity was defined as the amount of protein required to generate 1 μmol of p-nitrophenol per minute. Specific activity was expressed as units per milligram of protein. Protein concentrations were determined using the bicinchoninic acid (BCA) protein assay (Pierce, Rockford, IL), with bovine serum albumin (Sigma Aldrich, USA) as the standard. All assays were performed in triplicate. In comparative analysis of the GRAS host strains, the strain, which showed high enzymatic activity COMPARED with recombinant *E*. *coli* was selected for further experiments.

#### 2.4.3. Electrophoresis

SDS-PAGE analysis was performed using a 10% acrylamide-bis-acrylamide gel (37.5:1 [Qbiogene]). The culture samples were prepared by mixing dye with the samples (3:1) of each cell suspension. The solutions were mixed well and heated for 5 min at 100°C. Similarly, in each lane of the gel 15 μL of dye-sample mixture was loaded and electrophoresis was performed in SDS-Tris-Glycine buffer at a constant voltage until the dye front reached the bottom of the gel. The protein bands were stained with Coomassie brilliant Blue Ez stain (AQua), and de-stained in distilled water. After de-staining, the results of the GRAS host strains were compared with those of the recombinant *E*. *coli*. Based on the comparative analysis of GRAS hosts with recombinant *E*. *coli* strain, the highly expressed *β*-glucosidase enzyme of *C*. *glutamicum* was selected for biotransformation of the ginsenoside Rg_3_-Mix.

### 2.5. Biotransformation activity of Rg_3_-Mix using BglPm_C from C. glutamicum

To check the effect of fusion His tag on the activity of BglPm_C, the initial transformation of Rb_1_ and 20(*S*)-Rg_3_ show that the His tag-fused enzyme does not affect the activity of BglPm_C for conversion of ginsenosides Rb_1_ and 20(*S*)-Rg_3_ into F_2_ and 20(S)-Rh_2_, respectively (data not shown). Therefore, the His tag fusion protein (BglPm_C) was used for the hydrolysis of the glucose moieties attached at the C-3 sites in the Rg_3_-Mix [20(*S*)-Rg_3_, 20(*R*)-Rg_3_, Rk_1_, Rg_5_]. The enzyme (20 mg/ml) was reacted with Rg_3_-Mix solution at a concentration of 5% (w/v, wet base) in 100 mM of sodium phosphate buffer (pH 7.0) at 37°C. The samples were taken at regular intervals of time and analyzed via thin layer chromatography (TLC) or high performance liquid chromatography (HPLC) after pretreatment (see section 2.7).

### 2.6. Scaled-up biotransformation of Rg_3_-Mix into Rh_2_-Mix

#### 2.6.1. Preparation of recombinant enzyme (BglPm_C) of *C*. *glutamicum* using high cell density culture

For fed-batch cultivation and obtaining high cell density of the recombinant BglPm_C, the LB medium supplemented with kanamycin (50 mg/l final) was used to cultivate the *C*. *glutamicum* harboring pCES208 in a 10 L stirred-tank reactor (Biotron GX, Hanil Science Co., Korea) with a 6-L working volume at 400 rpm. Using 100 mM sodium phosphate buffer the pH value of the medium was adjusted to 7.0. The culture was incubated at 30°C for 24 h and the protein expression was induced through the addition of glucose with a final concentration of 10 g/l. After cell density reached an OD of 40–42 at 600 nm, the cells were harvested via centrifugation at 8,000 rpm for 20 min. The pellets (50 g) were resuspended in 100 mM of sodium phosphate buffer (pH 7.0); then the cells were broken via sonication (Branson Digital Sonifier, Mexico), and the time was adjusted according to the method described in section *2*.*4*.*1*. In order to get the crude soluble enzyme fraction for the conversion of ginsenosides, the unwanted cells debris was removed via centrifugation at 5,000 rpm for 10 min at 4°C. For the enzymatic biotransformation of ginsenoside Rg_3_-Mix, the crude recombinant BglPm_C was diluted to the desired concentration with 100 mM sodium phosphate buffer (pH 7.0) and was for the conversion of ginsenoside Rg_3_-Mix.

#### 2.6.2. Production of Rh_2_-Mix from Rg_3_-Mix using BglPm_C

For the mass production of ginsenoside Rh_2_-Mix, the reaction mixture was performed in a 10-L stirred-tank reactor (Biotron GX, Hanil Science Co.) with a 3-L of working volume. The reaction mixture was started with a composition of 50 mg/ml (final concentration) of substrate ginsenosides (Rg_3_-Mix; total 150 g, wet base) and 20 mg/ml of crude recombinant BglPm_C in 0.1 M of sodium phosphate buffer (pH 6.5–7.0). The reaction was completed under its optimal conditions of pH 6.5 with 300 rpm for 24 h. After 24 h, the ginsenoside Rg_3_-Mix was completely converted to the Rh_2_-Mix. Samples were collected at regular intervals and were analyzed by high performance liquid chromatography (HPLC) in order to determine the time course of the biotransformation of ginsenoside Rg_3_-Mix to Rh_2_-Mix.

### 2.7. Analytic methods

#### 2.7.1. TLC analysis

The ginsenosides spots on the TLC plates [60F254 silica gel plates (Merck, Germany)] were identified and visualize through comparisons with ginsenosides standard using CHCl3-CH3OH-H2O (65:35:10, lower phase) as TLC solvent and 10% (v/v) H_2_SO_4_ as a spraying reagent for spots visualization followed by heating of 110°C for 5–10 min.

#### 2.7.2. HPLC analysis

The ginsenosides PPD-Mix, Rg_3_-Mix, Rh_2_-Mix (by BglPm_C) and ginsenoside standards with a final concentration of 1 mg/ml were dissolved in HPLC grade methanol and analysed by HPLC (Younglin Co. Ltd, Korea). The ginsenosides separation was performed on a Prodigy ODS (2) C_18_ column (5 μm, 150 × 4.6 mm i.d.; Phenomenex, USA) with a guard column (Eclipse XDB C_18_, 5 μm, 12.5 × 4.6 mm i.d.). The mobile phases were water (line B) and acetonitrile (line C). The gradient elution started with 68% of solvent B and 32% of solvent C; the flow rate was 1.0 ml/min and detection was performed by monitoring the absorbance at 203 nm with an injected volume of 25 μl for 28 min.

## 3. Results and discussion

### 3.1. Cloning, expression and comparison of recombinant BglPm in different GRAS host strains

**Fig 1:**
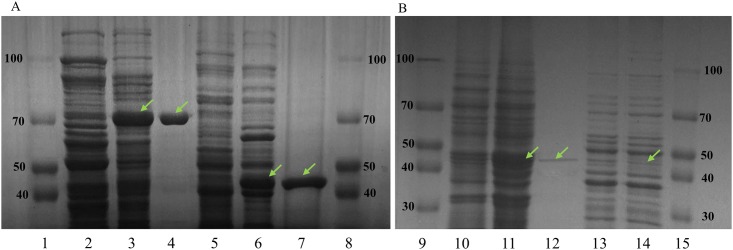
(A and B). SDS-PAGE analysis of recombinant E. coli and GRAS host strains. **A**: Lane 1, molecular weight standard; lane 2, soluble crude extract of recombinant *E*. *coli* without induction; lane 3, BglPm of recombinant *E*. *coli* after induction; lane 4, purified soluble fraction of recombinant *E*. *coli* (BglPm); lane 5, non-inducible fraction of *Corynebacterium glutamicum* harboring pCES208; lane 6, inducible BglPm_C; lane 7, purified BglPm_C (*C*. *glutamicum*); lane 8, molecular weight standard. **B:** lane 9, molecular weight standard; lane 10, non-inducible fraction of *Saccharomyces cerevisiae*; lane, 11 inducible BglPm_S; lane 12, BglPm_S protein of *S*. *cerevisiae* after purification; lane, 13–14, non-inducible and inducible fraction of *Lactococcus lactis*; lane 15, molecular weight standard.

The *β*-glucosidase gene consists of 1,260 bp and encodes 419 amino acids, with 46 kDa M.wt, which have homology to the protein domain of the glycoside hydrolase family 1. The gene (*β*-glucosidase) was amplified via PCR and then inserted into the pGEX 4T-1, pYES2.1, pCES208 and pNZ8148 vectors respectively. The predictive molecular masses and expression level of the recombinant BglPm were also determined by SDS-PAGE, and the protein expression of the GRAS host strains were compared with the recombinant *E*. *coli* system (Fig 1A and 1B). The molecular masses of the native *β*-glucosidase were calculated via an amino acid sequence and fusion tag protein found to be 72 (46+26), 47 (46+1), 47 (46+1), and 46 kDa (Table 3), as expressed by *E*. *coli*, *C*. *glutamicum*, *S*. *cerevisiae*, and *L*. *lactis*, respectively. The GST-BglPm and His-tag-BglPm were purified using the GST and His-tag bind resin column (Elpis Biotech). After purification of cell lysates, non-induced, induced, and purified protein soluble fractions were analyzed by SDS-PAGE and the prominent protein bands, with an apparent molecular weight near 72, 47, 47 and 46 kDa, were identified in three GRAS host strains and recombinant *E*. *coli* lysates. In the comparative study of SDS-PAGE assay of GRAS host strains with *E*. *coli* (Fig 1A, lane 3,4) it was clearly shown that the expected protein bands were more visible and well expressed in the soluble fraction in *C*. *glutamicum* (Fig 1A, lane 6,7) than in *S*. *cerevisiae* and *L*. *lactis* (Fig 1B, lane 11–12 and 13–14).

**Table 3:** Total activities of *β*-glucosidase in cell-free extracts of induced *E*. *coli* and GRAS host strains which were constructed with different vectors, and induced by different promoters used in this study. The GRAS strain, which was well-expressed and showed high enzyme activity with recombinant *E*. *coli*, is indicated in bold.

Hosts	Media	Vectors	Inducers	Fusion tags	M.wt of fusion tag protein/ recombinant protein (kDa)	Name of recombinant enzyme	Enzymes activity	Specific activity (U/mg)*	Relative express enzymes activities
*E*. *coli*	LBA	PGEX4T-1	IPTG	GST	26/46	BglPm	0.2619	10.22 ± 0.62	100
***C*. *glutamicum***	**LBK**	**pCES208**	**Glucose**	**His tag**	**1/46**	**BglPm_C**	**0.1976**	**12.54** ± 0.51	**75.4**
*S*. *cerevisiae*	YPD	pYES2.1	Galactose	His tag	1/46	BglPm_S	0.03	12.92 ± 0.13	11.5
*Lactococcus lactis*	M-17	pNZ8148	Nisin	–	–/46	BglPm_L	0.0244	ND	9.3

*One unit of activity was defined as the amount of protein required to generate 1 mmol of p-nitrophenol per min. Enzymes were purified ones.

Abbreviation; LBA (Luria-Bertani with ampicillin); LBK (Luria-Bertani with Kanamycin); YPD (Yeast+ peptone and dextrose); ND (not determined)

### 3.2. Effect of sonication on enzymes activities

**Fig 2:**
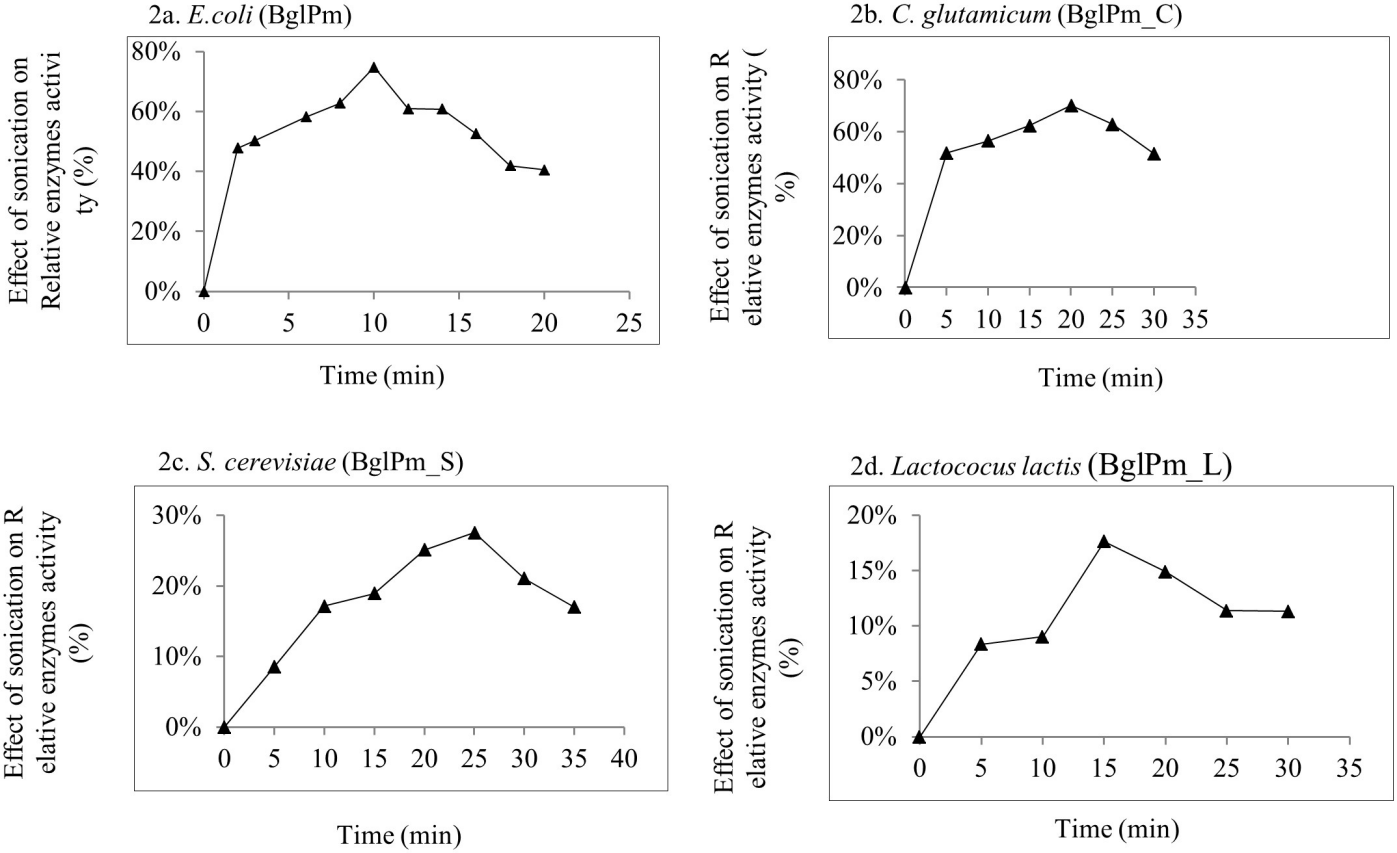
(a, b, c and d) shows the effect of sonication on the enzyme activity of; recombinant BglPm (*E*. *coli*), BglPm_C (*C*. *glutamicum*), BglPm_S (*S*. c*erevisiae*) and BglPm_L (*Lactococcus lactis*), respectively. From the sonication analysis, these results clearly show that enzymes lose their activities after a specific time interval for all recombinant enzymes used in this study.

Cell suspensions of recombinant *E*. *coli* and GRAS hosts were sonicated for different periods, ranging from 2 to 30 min, in 50-ml tubes. After the sonication, the cell lysates were separated into soluble and particulate fractions by centrifugation and each soluble fraction was assayed for its enzyme activity. During the investigation of enzymes activities of the GRAS host and recombinant *E*. *coli*, which were reacted with 5 mM pNPG, the maximum enzyme activity was obtained by recombinant *E*. *coli* after a 10 min period of sonication; further sonication caused loss of enzyme activity (Fig 2a). Comparable results were obtained from GRAS hosts, which showed optimum enzyme activity at 20, 25 and 15 min for *C*. *glutamicum* pCES208 (Fig 2b), *S*. *cerevisiae* pYES2.1 (Fig 2c), and *L*. *lactis* pNZ8148, respectively (Fig 2d). Collectively, these results suggest that the maximum enzyme activity of the GRAS host strains were compared with recombinant *E*. *coli*. On the basis of data presented here, we found that BglPm_C expressed by *C*. *glutamicum* had an enzyme activity of 75.4% COMPARED with recombinant BglPm expressed by *E*. *coli* (as compared to BglPm_S [11.5%] and BglPm_L [9.3%]), as shown in Table 3. We therefore selected highly expressed *β*-glucosidase (BglPm_C) by *C*. *glutamicum* for the mass production of edible Rh_2_-Mix ginsenosides from Rg_3_-Mix.

### 3.3. Biotransformation of Rg_3_-Mix to Rh_2_-Mix

**Fig 3:**
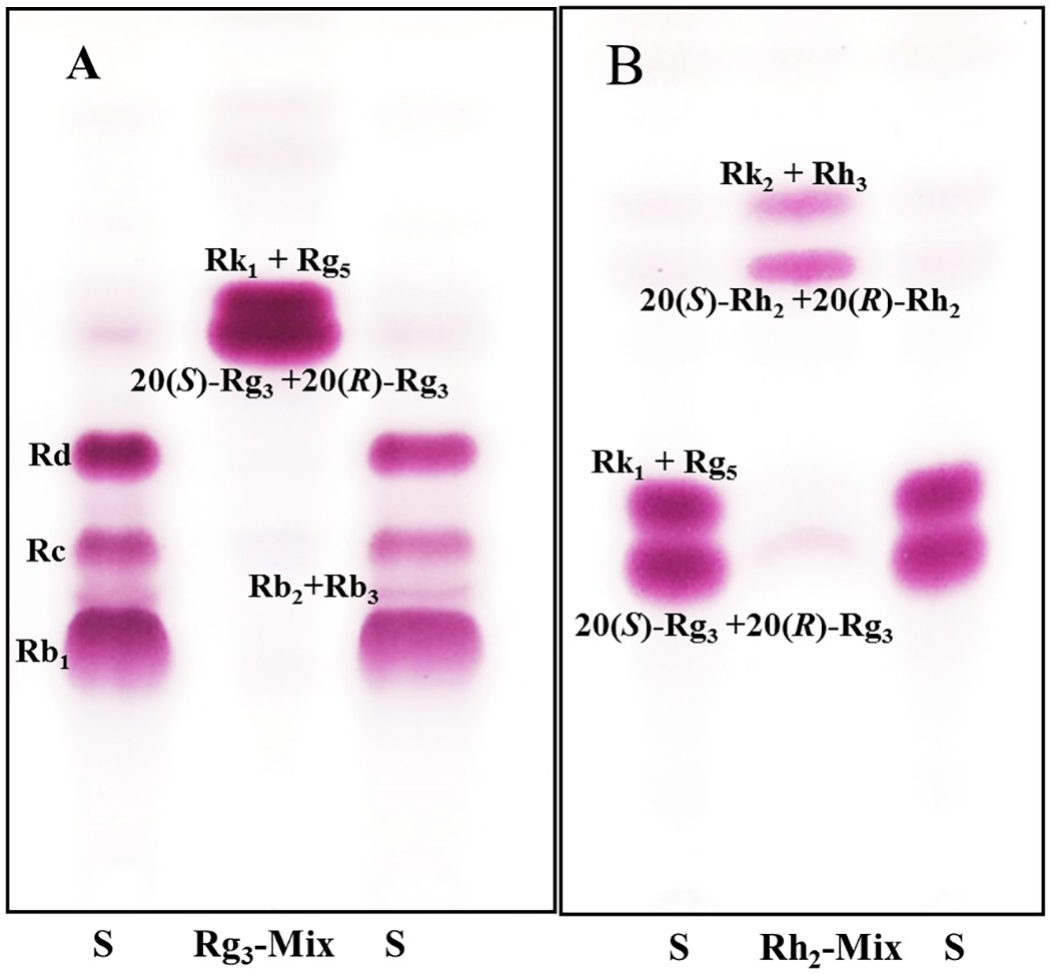
TLC analyses of time course of ginsenosides by acid and enzyme (BglPm_C) treatment. (A) Transformation of ginsenoside PPD-Mix. (B) Biotransformation of Rg_3_-Mix to Rh_2_-Mix after 24 h. Developing solvent: CHCl_3_-CH_3_OH-H_2_O (65:35:10, lower phase). Lane S represents PPD-Mix (A) and Rg_3_-Mix (B). PPD, protopanaxadiol.

To verify the bioconversion of Rg_3_-Mix by BglPm_C expressed by *C*. *glutamicum* harboring pCES208, TLC and HPLC analyses were carried out at regular intervals. TLC analysis show that BglPm_C completely transformed ginsenosides Rg_3_-Mix into Rh_2_-Mix, as shown in Fig 3A and 3B. The Rf values of ginsenoside Rk_1_ and Rg_5_ was a little above the 20(*S*)-Rg_3_ and 20(*R*)-Rg_3_ position as shown in Fig 3A. Rh_2_-Mix, which has one glucose moiety removed at the C20 position of Rg3-Mix, was placed in the upper position of control S (Rg_3_-Mix), as shown in the middle position of Fig 3B.

### 3.4. Preparation of BglPm_C and scaled-up production of Rh_2_-Mix as a gram unit

**Fig 4:**
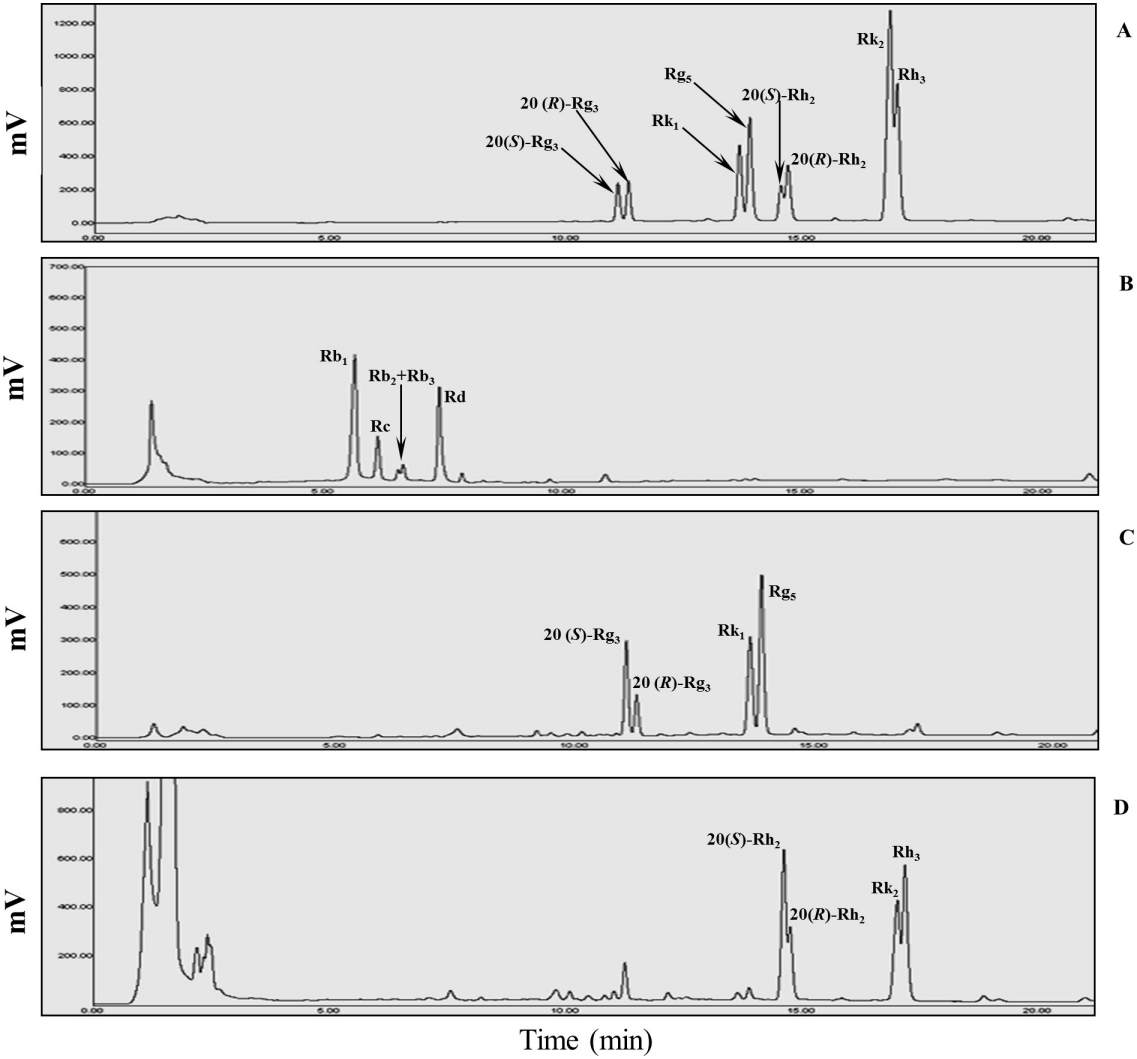
HPLC analysis of the transformation of the ginsenosides (PPD-Mix and Rg_3_-Mix) by acid and enzyme treatments. (A) Ginsenosides standard. (B) PPD-Mix as a starting substrate. (C) Rg_3_-Mix after 15 min at 121°C by acid treatment of PPD-Mix. (D) Rh_2_-Mix after 24 h of the reaction of BglPm_C with Rg_3_-Mix. PPD-Mix, protopanaxadiol-type ginsenoside mixture (Rb_1_, Rb_2_, Rb_3_, Rc and Rd).

The *C*. *glutamicum* cells that harbor pCES208 were further incubated for 24 h at 30°C and induced by the addition of 10 g/l of glucose. After induction, when the culture reached an OD of 40–42 at 600 nm the cells were harvested via centrifugation. 100 g of wet cells were harvested and resusupended (50 g/500 ml [w/v]) in 0.1 M of phosphate buffer (pH 7.0) (w/v). The cells were broken via ultrasonication and the supernatant was used as crude enzymes for the biotransformation of the ginsenoside Rg_3_-Mix. The crude recombinant BglPm_C (soluble form) was applied to the biotransformation reactor. The enzyme reaction was induced using the crude recombinant BglPm_C, which was adjusted to a final concentration of 20 mg/ml in a 3-L tank, to produce the Rh_2_-Mix. The ginsenoside Rg_3_-Mix [20(*S*)-Rg_3_, 20(*R*)-Rg_3_, Rk_1_, and Rg_5_] was completely converted to Rh_2_-Mix [20(S)-Rh_2_, 20(R)-Rh_2_, Rk_2_, and Rh_3_]. After 24 h, the results were confirmed by HPLC analysis; all the ginsenosides (PPD-Mix, Rg_3_-Mix, and Rh_2_-Mix) were compared with the ginsenosides standards that were used in this study, as shown in Fig 4A. The PPD-Mix type ginsenoside (Fig 4B) was used as the initial substrate. For the enzymatic reaction, the PPD-Mix was transformed to the Rg_3_-Mix by acid treatment as shown in Fig 4C. Lastly, after 24 h, the Rh_2_-Mix [20(*S*)-Rh_2_, 20(*R*)-Rh_2_, Rk_2_ and Rh_3_] was produced as a final product from the bioconversion of Rg_3_-Mix using the BglPm_C enzyme of *C*. *glutamicum* (Fig 4D). The HPLC analysis revealed that the BglPm_C completely hydrolyzed the Rg_3_-Mix within 24 h.

In order to remove the unwanted substances, the reaction mixture was centrifuged at 8,000 rpm for 10 min. Most of the ginsenoside Rh_2_-Mix precipitated to form a solid, with a small quantity remaining dissolved in the supernatant (data not shown). Three liters of a 95% ethanol solution was used, twice, to dissolve the precipitated ginsenosides Rh_2_-Mix thoroughly. The ginsenosides Rh_2_-Mix in the supernatant was evaporated *in vacuo* in order to create 24.5 g of powdered Rh_2_-Mix [20(*S*)-Rh_2_ (116.6 mg/g), 20(*R*)-Rh_2_ (107.2 mg/g), Rk_2_ (143.1 mg/g) and Rh_3_ (165.0 mg/g)]. Finally, in terms of yield, 24.5 g of Rh_2_-Mix was obtained via the conversion of 50 g of PPD-Mix as the initial substrate S1 Fig.

In oriental herbal medicine, red ginseng is a very popular health-promoting food but contains approximately less than 0.02% Rh_2_ or Rh_2_-Mix based on dry weight. Although Rh_2_ has anti-cancer effect for breast cancer, prostate cancer, hepatoma, gastric cancer, colon carcinoma, and learning and memory effects, the lack of a selective mass-production technology has hindered its commercial uses. To get a scaled up production of Rh_2_-Mix, a number of researchers sought to achieve biotransformation of major ginsenosides to minor ginsenosides using microorganisms [34] and recombinant enzymes in laboratory settings [35, 36, 41]. Ko et al [36] were only able to obtain a 10 mg scale of Rg_2_(S) and Rh_1_(S) from the bioconversion of PPT-type ginsenosides using crude *β*-galactosidase from *Aspergillus oryzae* and crude lactase from *Penicillium* sp. Similarly, Juan et al. produced ginsenoside Rg_2_(S) as a 100-gram unit using recombinant *β*-glucosidase from *Pseudonocardia* sp. Gsoil 1536 [30]. Despite the use of recombinant enzymes, no study has yet focused on the mass production of minor ginsenosides using edible enzymes from GRAS host strains.

**Fig 5:**
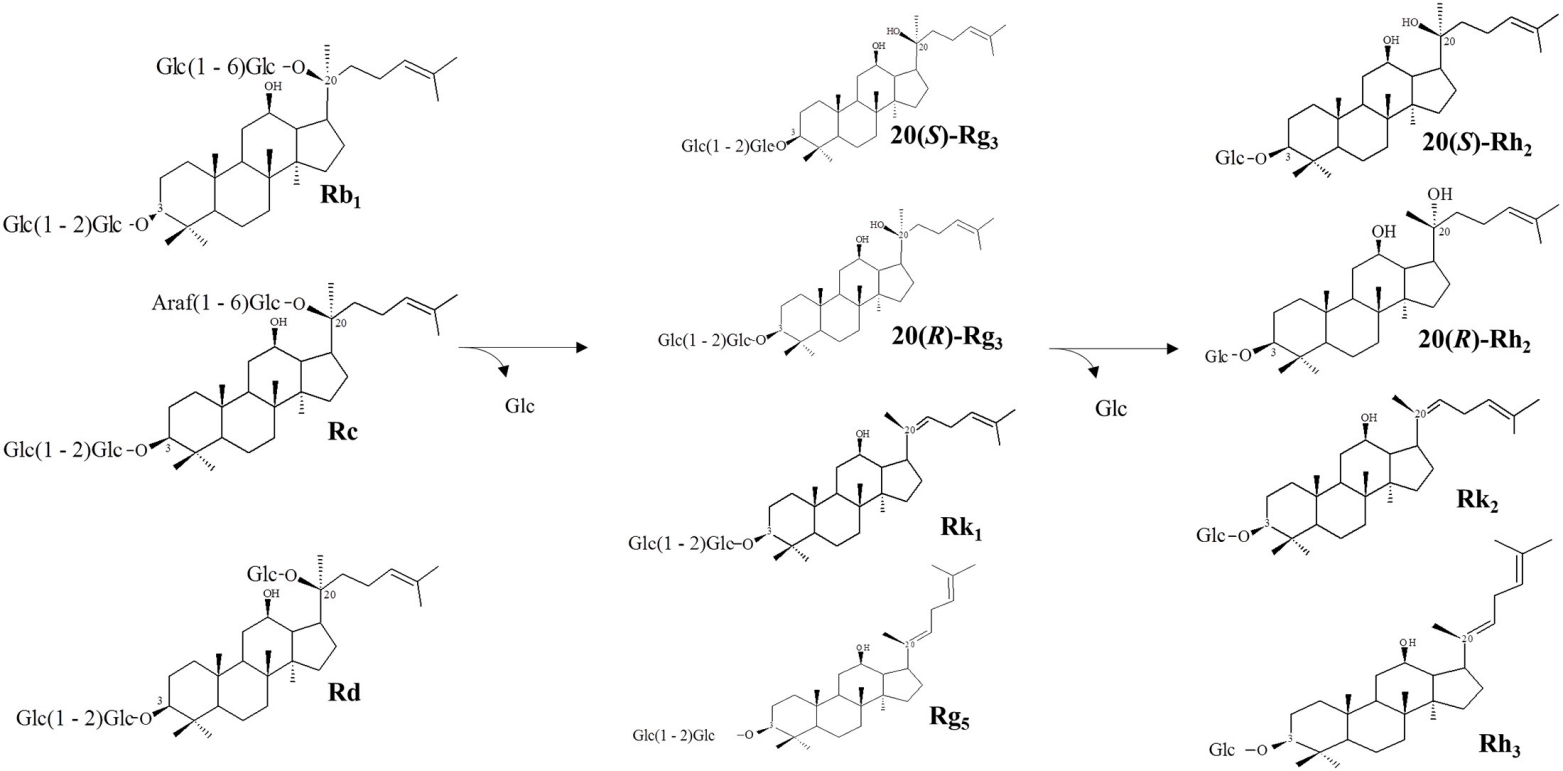
Schematic view of transformation pathways for Rh_2_-Mix production and the relative structures of ginsenosides.

In this study, we describe a brief comparison between GRAS host strains and recombinant *E*. *coli* and the effect of sonication on enzyme activity of GRAS hosts and *E*. *coli*. Furthermore, we found a BglPm_C, comparable to *E*. *coli*, from *C*. *glutamicum*, which is capable of the biotransformation of Rg_3_-Mix and is therefore expected to facilitate the mass production of Rh_2_-Mix. The BglPm_C shows a very specific ginsenoside hydrolysis activity via the following pathways: 20(*S*)-Rg_3_→20(*S*)-Rh_2_, 20(*R*)-Rg_3_→20(*R*)-Rh_2_ and ginsenoside Rk_1_→RK_2_ and Rg_5_→Rh_3_ as shown in Fig 5. This unique bioconversion ability, together with optimum reaction conditions (30°C and pH 6.5/7.0) [27], makes it possible to produce 24.5 gram-scale Rh_2_-Mix. Here, we report for the first time that the BglPm_C expressed by one of GRAS hosts (*C*. *glutamicum*) can be used to make up to 50 mg/ml of PPD-Mix to 24.5 gram-scale Rh2-Mix within 24 h. This combined treatment of acid and the use of the recombinant enzyme BglPm_C expressed in *C*. *glutamicum* enable the usage of ginsenoside Rh_2_ and Rh_2_-Mix derived from *Panax quinquefolius* (American ginseng) or *Panax ginseng* Meyer (Korean ginseng) in the cosmetics, functional food, and pharmaceutical industries by replacing the *E*. *coli* expression system.

## Conclusion

Upon choosing appropriate experimental organisms for *β*-glucosidase gene expression, we found alternative systems using food grade bacteria for the expression of *β*-glucosidase gene rather than recombinant *E*. *coli*. By means of BglPm_C, 24.5 g of ginsenoside Rh_2_-Mix was achieved via biotransformation of 50 g of PPD-Mix initial substrate consisting of ginsenosides Rb_1_, Rb_2_, Rb_3_, Rc, and Rd. The bioconversion reaction was started in a 10 L jar fermenter at pH 6.5 and 30°C for 24 h, with a substrate concentration of 50 mg (Rg_3_-Mix, wet base). This combinational usage of edible organic acid treatment of PPD-Mix to Rg_3_-Mix and enzymatic transformation of Rg_3_-Mix to Rh_2_-Mix offers an efficient method for the preparation of minor ginsenoside Rh_2_-Mix on a large scale to meet industrial needs.

## Supporting information

S1 Fig Entire process of Rh_2_-Mix production from PPD-Mix (protopanaxadiol-type ginsenoside) as starting substrate using combined method of acid heat treatment and enzyme treatment (BglPm_C). (TIF)
