# Supplementary figures and images for: Comparative analysis of the expression level of recombinant ginsenoside-transforming β-glucosidase in GRAS hosts and mass production of the ginsenoside Rh2-Mix
Source: PLoS One. 2017 Apr 19;12(4):e0176098. doi: 10.1371/journal.pone.0176098 (PMC5396970; doi:10.1371/journal.pone.0176098)

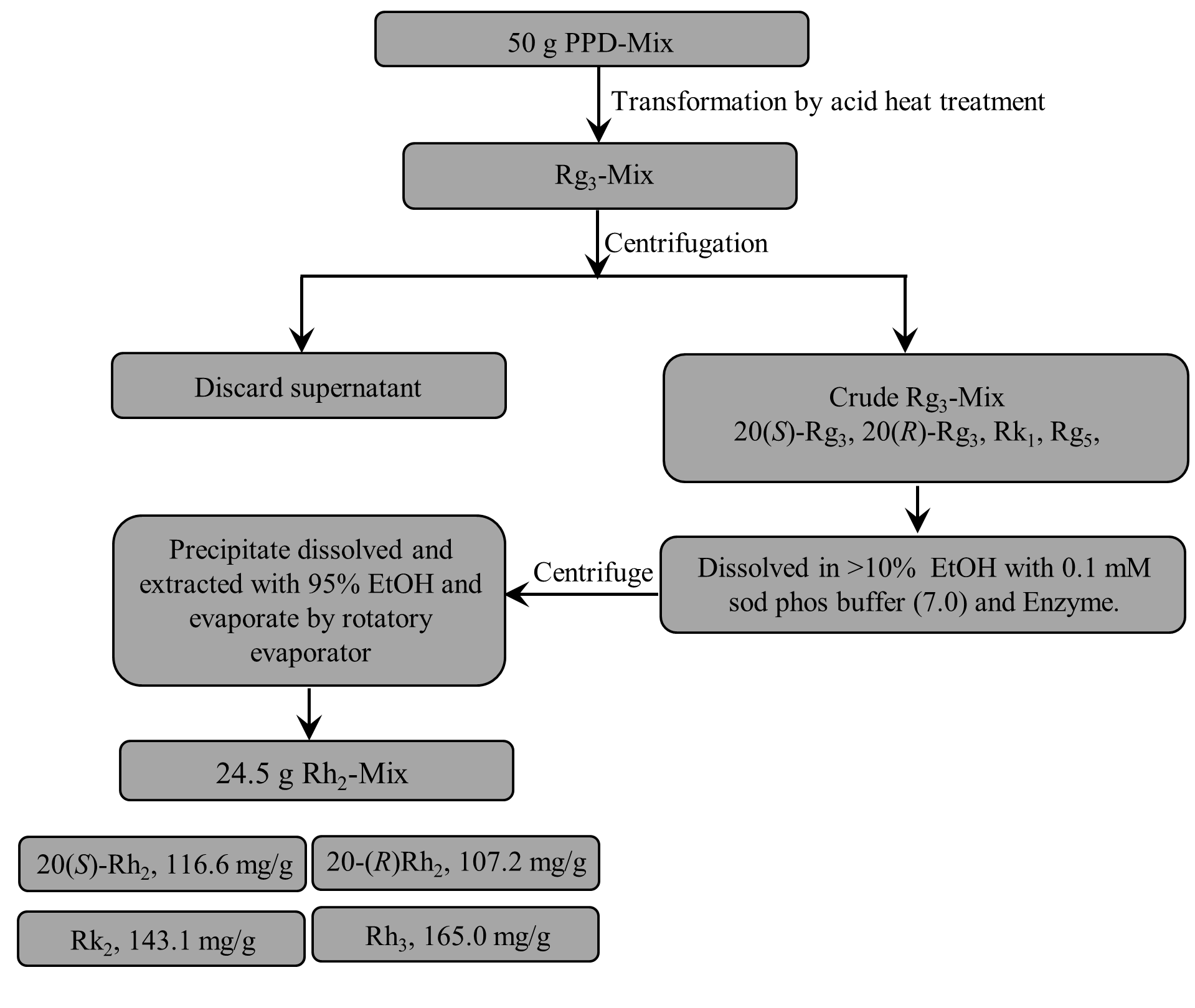

Supplement: S1 Fig — (TIF) [file pone.0176098.s001.tif]
